# Expression and Stability of Foreign Epitopes Introduced into 3A Nonstructural Protein of Foot-and-Mouth Disease Virus

**DOI:** 10.1371/journal.pone.0041486

**Published:** 2012-07-27

**Authors:** Pinghua Li, Xingwen Bai, Yimei Cao, Chenghao Han, Zengjun Lu, Pu Sun, Hong Yin, Zaixin Liu

**Affiliations:** State Key Laboratory of Veterinary Etiological Biology, National Foot and Mouth Disease Reference Laboratory, Lanzhou Veterinary Research Institute, Chinese Academy of Agricultural Sciences, Lanzhou, Gansu, China; Institut Pasteur, France

## Abstract

Foot-and-mouth disease virus (FMDV) is an aphthovirus that belongs to the *Picornaviridae* family and causes one of the most important animal diseases worldwide. The capacity of other picornaviruses to express foreign antigens has been extensively reported, however, little is known about FMDV. To explore the potential of FMDV as a viral vector, an 11-amino-acid (aa) HSV epitope and an 8 aa FLAG epitope were introduced into the C-terminal different regions of 3A protein of FMDV full-length infectious cDNA clone. Recombinant viruses expressing the HSV or FLAG epitope were successfully rescued after transfection of both modified constructs. Immunofluorescence assay, Western blot and sequence analysis showed that the recombinant viruses stably maintained the foreign epitopes even after 11 serial passages in BHK-21 cells. The 3A-tagged viruses shared similar plaque phenotypes and replication kinetics to those of the parental virus. In addition, mice experimentally infected with the epitope-tagged viruses could induce tag-specific antibodies. Our results demonstrate that FMDV can be used effectively as a viral vector for the delivery of foreign tags.

## Introduction

Foot-and-mouth disease virus (FMDV) is the causative agent of foot-and-mouth disease (FMD), a highly contagious and economically important disease of cloven-hoofed animals, including cattle, swine, goats, sheep and other species of wild ruminants. The virus belongs to the *Aphthovirus* genus within the family *Picornaviridae* and has a single-stranded positive-sense RNA genome approximately 8.5 kb in length. The 1300-nt 5′ untranslated region (5′ UTR) is followed by a single long open reading frame (ORF), the 3′ untranslated region (3′ UTR), and Poly (A) tail. The ORF encodes four structural proteins (VP1, VP2, VP3, and VP4) and several precursors, as well as a total of nine mature nonstructural proteins. Each of these nonstructural proteins is involved in multiple functions needed for RNA genome replication and particle formation in infected cells [1], [2].

FMDV can be differentiated from other picornaviruses by a longer 3A protein (153 aa instead of 87 aa for poliovirus) and three copies of 3B. Although 3A has been found to be physically associated with intracellular membranes that proliferate in picornaviruses infected cells [3], [4], [5], [6], the functions of nonstructural protein 3A in the life cycle of FMDV is less well understood. However, alterations in 3A protein, such as point mutations and deletions, were linked to altered host specificity, adaptation, attenuation and virulence of FMDV [7], [8], [9], [10], [11], [12].

The potential for infectious cDNA clones to act as foreign gene expression vectors has been extensively explored for both negative-stranded RNA viruses and positive-stranded RNA viruses in recent years. A number of recombinant RNA viruses expressing various foreign genes were stably maintained during serial passages, indicating the suitability of these viruses for the development of viral vectors [13], [14], [15]. For example, the green fluorescent protein (GFP) gene was inserted between the fusion (F) and hemagglutinin-neuraminidase (HN) genes of Newcastle disease virus (NDV), leading to an infectious particle maintained stably for at least five passages in embryonated eggs [16]. A recombinant measles virus (MV) containing the hepatitis B virus (HBV) small surface antigen (HBsAg) was uniformly expressed after 10 serial passages [17]. The full-length PRRSV clone containing a foreign sequence coding for a total of 31 amino acids produced an infectious progeny virus, and this virus retained its infectivity and genetic stability during four passages of examination [18]. Considerable efforts also have gone into developing a variety of recombinant picornaviruses (including enterovirus [19], [20], cardiovirus [21], [22], rhinovirus [23] and hepatovirus [24]) that present heterologous immunogens on their surfaces. In particular, poliovirus (PV) has been reported extensively in this regard [25], [26], [27], [28], [29], and it has been shown that PV can serve as an effective vector for expressing foreign proteins. For FMDV, more recently, the possibility for generation of recombinant FMDVs carrying foreign epitopes has been shown by insertion of foreign tags between the inter-AUG regions [30]. However, to date, no studies have further reported the feasibility of FMDV as a viral vector for expressing foreign antigens.

In the present study, an 8-aa FLAG epitope (DYKDDDDK) and an 11-aa HSV epitope (QPELAPEDPED) derived from herpes simplex virus (HSV) glycoprotein D were introduced into the C-terminal different regions of 3A protein of an FMDV full-length infectious cDNA clone to construct recombinant viruses. We demonstrated that infectious virus expressing FLAG or HSV tag protein could be produced and the 3A-tagged viruses shared similar plaque phenotypes and replication kinetics to those of the parental virus. Furthermore, the FLAG and HSV epitopes were stably maintained and expressed even after 11 serial passages in BHK-21 cells. In addition, FLAG/HSV-specific antibodies could be induced after inoculation the 3A-tagged viruses in mice. These observations indicate that FMDV can be used effectively as a viral vector for the delivery of foreign epitope tags.

## Materials and Methods

### Cells and Virus

Baby hamster kidney cells (BHK-21) [31] were grown in Dulbecco’s modified Eagle’s medium (DMEM) supplemented with 10% FCS. BSR-T7/5 cells [32], which stably express T7 RNA polymerase, were provided by K. K. Conzelmann (Max-von-Pettenkofer Institut, Munich, Germany) and were grown in Glasgow minimal essential medium (GMEM) supplemented with 10% FCS and 1 mg/ml G418. All cells were grown at 37°C in a humidified chamber containing 5% CO_2_. The parental virus (r-HN) used in this study was recovered from BSR-T7/5 cells transfected with *Not* I-linearized plasmid pOFS, which contained the complete FMDV O/HN/CHA/93 genoe. The properties of this virus have been described previously [33]. The virus was titrated on BHK-21 cells by calculating the 50% tissue culture infectious dose per ml (TCID_50_/ml).

### Antibodies

Anti-FMDV 3A protein mouse monoclonal antibodies were generated as described previously [34]. Mouse monoclonal anti-HSV antibody was purchased from Novagen and mouse monoclonal anti-FLAG antibody was purchased from Sigma. Fluoresceinisothiocyanate (FITC)-conjugated goat anti-mouse secondary antibody used in immunofluorescence and horseradish peroxidase (HRP)-conjugated goat anti-mouse secondary antibody used in western blot analyses were from Sigma.

### Introduction of Foreign Tags into the FMDV Full-length Infectious cDNA Clone

Plasmid pOFS for recovery of r-HN has been described previously [33] and this clone was used as the genomic backbone for the generation of mutant full-length plasmids. To construct recombinant FMDVs that allow the cloning of foreign genes in frame to the C-terminal region of FMDV 3A, overlap extension PCR was used. Briefly, two independent cDNA fragments containing FLAG nucleotide sequences were amplified using HN-1F/HN-1R and HN-2F/HN-2R primer pairs (Table 1). PCR amplifications were performed using PrimeSTAR HS DNA Polymerase (Takara). Cycling conditions for both PCRs were as follows: 94°C for 1 min followed by 30 cycles of 98°C for 20 s, 68°C for 1 min, and then 72°C for 8 min. These fragments were combined and subsequently amplified using the flanking primers HN-1F and HN-2R (Table 1). Reaction conditions included an initial denaturation at 94°C for 1 min, followed by 30 cycles of 98°C for 20 s, 68°C for 1.5 min, and a final step at 72°C for 8 min. Then, the fused fragment was digested with *Bgl* II and *Nru* I restriction enzymes and cloned into the *Bgl* II- and *Nru* I-digested fragment of pOFS to yield plasmid pOFS-FLAG. Similarly, plasmid pOFS-HSV was constructed using the HN-1F/HN-3R and HN-3F/HN-2R primer pairs (Table 1). Standard cloning procedures were performed as described by Sambrook et al. [35]. All constructs were verified by nucleotide sequencing to ensure that no other mutations had occurred during the cloning process.

**Table 1:** Primers used for the construction of genetically modified full-length clones of FMDV.

Primer	Sequence^a^(5′→3′)	Nucleotide Position
HN-1F	CAAGAAGTGATTGAGCGGGT	5274–5293
HN-1R	*CTTATCGTCATCGTCCTTATAGTC* CTGGCGCCTCTTGCGCGCTT	5683–5702
HN-2F	*GACTATAAGGACGATGACGATAAG* GACGCGGCTCTTGACGATGC	5613–5632
HN-2R	GCGTTGTTGATCACGCCGAC	6324–6343
HN-3F	*CAGCCGGAACTGGCTCCGGAAGACCCGGAAGAC* GTGCCCGGGAGGGAACAA	5736–5755
HN-3R	*GTCTTCCGGGTCTTCCGGAGCCAGTTCCGGCTG* TTGCTCGGTGGGGGATCT	5683–5702
HN-4F	AGGTGTCGAGTCACCCGATT	5308–5327
HN-4R	GTTCCCTTCTTCATTCTCGC	6297–6316

a Nucleotides encoding the FLAG and HSV tags are marked with italic type.

### Transfection and Recovery of Infectious Recombinant Viruses

Plasmids pOFS-FLAG and pOFS-HSV were linearized with *Not* I restriction site located downstream of the FMDV poly(A) sequence, and then purified using a QIAquick PCR Purification Kit (Qiagen). Confluent BSR-T7/5 cell monolayers (4–6×10^6^ in a six-well plate) were transfected with linearized plasmid DNAs using Lipofectamine™ 2000 (Invitrogen), according to the manufacturer’s protocol. After 5 h of incubation at 37°C, the supernatants were removed and replaced with GMEM supplemented with 4% tryptose phosphate broth and 10% FBS, and the cells were further incubated at 37°C. The BSR-T7/5 cell monolayers showed cytopathic effects (CPE) at 2 days post-transfection and the virus-containing supernatants were collected and titrated on BHK-21 cells.

### Immunofluorescence Assays (IFA)

BHK-21 cells (2×105) were seeded in a six-well plate, incubated for 12 h, and then mock-infected or infected with r-HN or the transfected supernatants at a multiplicity of infection (MOI) of 1. After 6 hours post-infection (hpi), the cells were washed three times with PBS and fixed with 4% paraformaldehyde (diluted in PBS) for 20 min at room temperature. The cells were washed three times and permeabilized with PBS containing 0.5% Triton X-100 (PBS-T) for 20 min. Thereafter, the cells were washed three times and blocked with blocking buffer (PBS, 10% bovine serum albumin) for 1 h. After further washing with PBS-T, the cells were immunostained with primary antibody (mouse anti-HSV tag mAb (1∶2000) or mouse anti FLAG-tag mAb (1∶1000) or 3 A mAb (1∶100)) for 1 h. The cells were rinsed with PBS-T and incubated with secondary antibody FITC-conjugated goat anti-mouse IgG antibody (1∶100) for 1 h. The cells were washed again with PBS-T and visualized using an Olympus BX40 fluorescence microscope.

### Western Blot Analysis

Confluent BHK-21 cells were mock-infected or infected with r-HN or the transfected supernatants at a MOI of 1. After 12 hpi, cells were washed with PBS, scraped, collected by low-speed centrifugation, and lysed with lysis buffer (50 mM Tris-HCl (pH 7.5), 150 mM NaCl, 1 mM EDTA, 1% Nonidet P-40, 0.1% sodium deoxycholate, and 25 mg of aprotinin per ml) for 30 min on ice. The cell lysates were clarified by centrifugation at 7,000×g for 10 min and the supernatants were mixed with Laemmli sample buffer (Qiagen) and boiled for 6 min before electrophoresis. The boiled samples were separated by 12% SDS-polyacrylamide gel electrophoresis (SDS-PAGE), and the resolved proteins were transferred to PVDF membranes (Sigma). Then, the membranes were blocked by incubation in PBS containing 5% nonfat milk for overnight at 4°C. After washing four times with PBS-T, the membranes were incubated with mouse anti-HSV tag mAb (1∶2,000) or mouse anti –FLAG tag mAb (1∶1000), respectively. Following four washes in PBS-T, the membranes were incubated with HRP-conjugated goat anti-mouse antibody (1∶1000). Following four additional washes with PBS-T, the proteins were visualized after further incubation with diaminobenzidine (DAB).

### Identification of Recombinant Viruses by RT-PCR

Viral RNAs were extracted from the transfected supernatants using a QIAamp Viral RNA Mini kit (Qiagen) and were used as template for RT-PCR analysis of the 3A gene of the recombinant viruses. RTs were performed with reverse transcriptase XL (AMV) at 42°C for 60 min, using HN-2R primer. After the reactions were completed, the enzyme was inactivated by incubation at 100°C for 3 min. PCRs were carried out with primer HN-1F and HN-2R. PCR reaction conditions included an initial denaturation at 94°C for 1 min, followed by 30 cycles of 98°C for 20 s, 68°C for 1.5 min, and a final step at 72°C for 8 min. The resulting PCR fragments of 1050 bp were purified and sequenced to confirm that the rescued viruses were the expected recombinants.

The transfected supernatants were serially passaged in BHK-21 11 times to determine the stability of the introduced modifications. Briefly, confluent BHK-21 cell monolayers (4–6×10^6^ in 100-mm-diameter dishes) were infected with the transfected supernatants at a MOI of 1. After 1 hpi, the inoculums were removed and cells were washed with PBS to remove unattached viruses. Then, 10 ml of DMEM supplemented with 2% FCS and antibiotics, was added to the dishes and incubated at 37°C in 5% CO_2_. The supernatants were collected until about 95% of the cells showed CPE (about 8–12 h). Each supernatant was serially passaged for further experiment. Viral RNAs were extracted from viral suspensions of the 4th, 8th and 11th passages and were subjected to RT-PCR analysis as described above. The stability of the recombinant viruses (p11) expressing foreign epitopes was further confirmed by IFA and Western blot analysis.

### Growth Curve Analysis and Plaque Morphology

One-step growth curves were performed for each virus in BHK-21 cells. Briefly, BHK-21 cell monolayers in 6-well plates were infected with r-HN or recombinant viruses at a MOI of 1 and incubated at 37°C in 5% CO_2_. After 1 h adsorption at 37°C, supernatants were removed and cell monolayers were washed twice with phosphate-buffered saline (PBS, PH 7.4). Thereafter, 2-ml DMED containing 2% FBS were added, and all monolayers were incubated at 37°C. At indicated times post-infection, cells were harvested and viruses were released from the cells by one freeze/thaw cycle, and the titer of the clarified supernatant was determined by TCID_50_ as described above. Plaque morphologies of the recombinant viruses and the parental virus were compared by plaque assay on BHK-21 cells as described previously [36].

### Infection of Mice

Six-week-old female Kunming white mice (n = 22) were purchased from the Laboratory Animal Center of Lanzhou Veterinary Research Institute, Chinese Academy of Agricultural Sciences. All mice were randomly divided into four groups. Groups 1 to 3 of 6 animals each were intraperitoneally inoculated with 1 ml cell culture medium containing 10^6^ TCID_50_ of each the 3A-tagged viruses or the parental virus. Group 4 with 4 animals were mock infected with PBS. The mice were bled retroorbitally at 28 day post-inoculation and serums were collected for analysis of anti-tag and anti-3ABC antibodies.

### Detection of Anti-tag Antibodies in Mice

Antibody responses to foreign tags were analyzed by testing each serum sample in an indirect enzyme-linked immunosorbent assays (ELISAs). Briefly, ELISA plates were coated with 3×FLAG or 3×HSV peptide antigen (Sigma) at a concentration of 2 µg/ml diluted in carbonate buffer (pH 9.6) and incubated overnight at 4°C. Plates were washed four times with PBS-T, and then blocked in PBS with 5% non-fat dry milk for 1 h. The plates were washed four times with PBS-T, and then 100 µl of diluted serum samples (1∶20 dilution in blocking buffer) were added. Serum samples were incubated for 1 h at 37°C, and then the plates were washed four times with PBS-T. A HRP-conjugated goat anti-mouse immunoglobulin G (1∶100 dilution in blocking buffer) was then added and incubated for 1 h at 37°C. After washing four times with PBS-T, the plates were incubated with O-Phenylendiaminedihydrochloride and stopped by adding 2 M H_2_SO_4_. Plates were read at a wavelength of 450 nm using an ELISA microplate reader. Samples were considered to be positive for tag antibodies if the ratios of the OD value of infected sera to mean negative control sera were greater than 2.1. The anti-3ABC antibody in each serum sample was also detected using a 3ABC-ELISA kit as previously described [37]. The sample value ≥0.2 was considered to be positive for 3ABC antibodies [37].

All animal studies were approved by the Review Board of Lanzhou Veterinary Research Institute, Chinese Academy of Agricultural Sciences (Permission number: SYXK-GAN-2004-0005). All animals used in the present study were humanely bred during the experiment and euthanasia was carried out at the end of the experiment.

## Results

### Construction and Recovery of Recombinant Viruses

**Figure 1:**
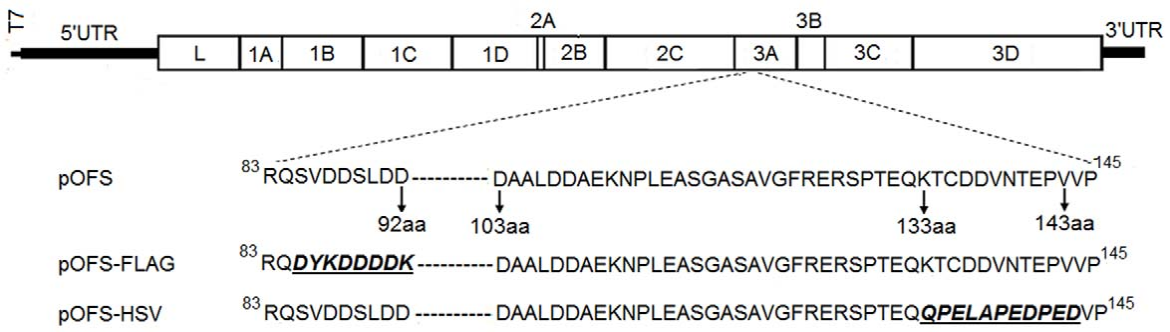
Schematic diagram of the FMDV genome and plasmids used in this study. The FMDV full-length genome cDNAs in these plasmids derived from the infectious cDNA clone, pOFS, were preceded by a synthetic T7 RNA polymerase promoter. The mutant plasmid pOSF-FLAG was produced by substituting native amino acid of the 85–92 region of the 3A protein for FLAG tag sequence and pOFS-HSV was produced by substituting native amino acids of the 133–143 region of the 3A protein for HSV tag sequence. Amino acid sequences of the tag are underlined. UTR: non-coding region. (-) indicates a naturally absent sequence.

Previous studies showed that PV can tolerate small insertions in the 3A gene to generate infectious viruses [38], [39]. It was not clear whether lethality was due to introduction foreign epitope tags into the 3A gene of FMDV. To investigate the potential of FMDV as a viral vector to express small tags, we introduced epitope sequences into different regions of 3A protein of an FMDV full-length infectious cDNA clone. As shown in Figure 1, two mutant plasmids pOFS-FLAG and pOFS-HSV were constructed. Plasmid pOFS-FLAG encodes the full-length genome of FMDV with a substitution of FLAG-tag sequence (DYKDDDDK) for native amino acid sequences (SVDDSLDD) of 85–92 aa of 3A protein. Plasmid pOFS-HSV encodes the full-length genome of FMDV with a substitution of HSV-tag sequence (QPELAPEDPED) for native amino acid sequences (KTCDDVNTEPV) of 133–143 aa of 3A protein. Viable viruses, named FMDV-FLAG and FMDV-HSV, were rescued when the corresponding plasmids were transfected into BSR-T7/5 cells. The results indicated that the C-terminal portion of FMDV 3A could tolerate small substitutions without effecting generation of viable viruses. Transfection supernatants were collected and serially passaged in BHK-21 cells for subsequent experiments. The characterization and titers of rescued viruses are summarized in Table 2.

**Table 2:** **Characterization of the recombinant viruses obtained by reverse genetics.**

		4th passage		8th passage		11th passage	
Construct^a^	Time of appearing CPE^b^	Retainedinsert^c^	Virus titer^d^TCID_50_/ml)	Retainedinsert^c^	Virus titer^d^(TCID_50_/ml)	Retainedinsert^c^	Virus titer^d^(TCID_50_/ml)
pOFS-HSV	48 h	yes	4×10^6^	yes	5.6×10^7^	yes	5×10^7^
pOFS-FLAG	48 h	yes	2.7×10^6^	yes	3.2×10^7^	yes	3.4×10^7^
pOFS	48 h	ND^e^	2.3×10^6^	ND^e^	3.4×10^7^	ND^e^	3×10^7^

a Three independent plasmid transfections were performed for each construct followed by passaging in BHK-21 cells.

b Time (h) when CPE was detected after transfection.

c The tag inserts were determined by RT-PCR and sequencing analysis.

d The virus titer were determined on BHK-21 cells by calculating the 50% tissue culture infectious dose per ml (TCID_50_/ml).

e ND: not done.

### Analysis of Foreign Tag Expression in Cell Cultures

**Figure 2:**
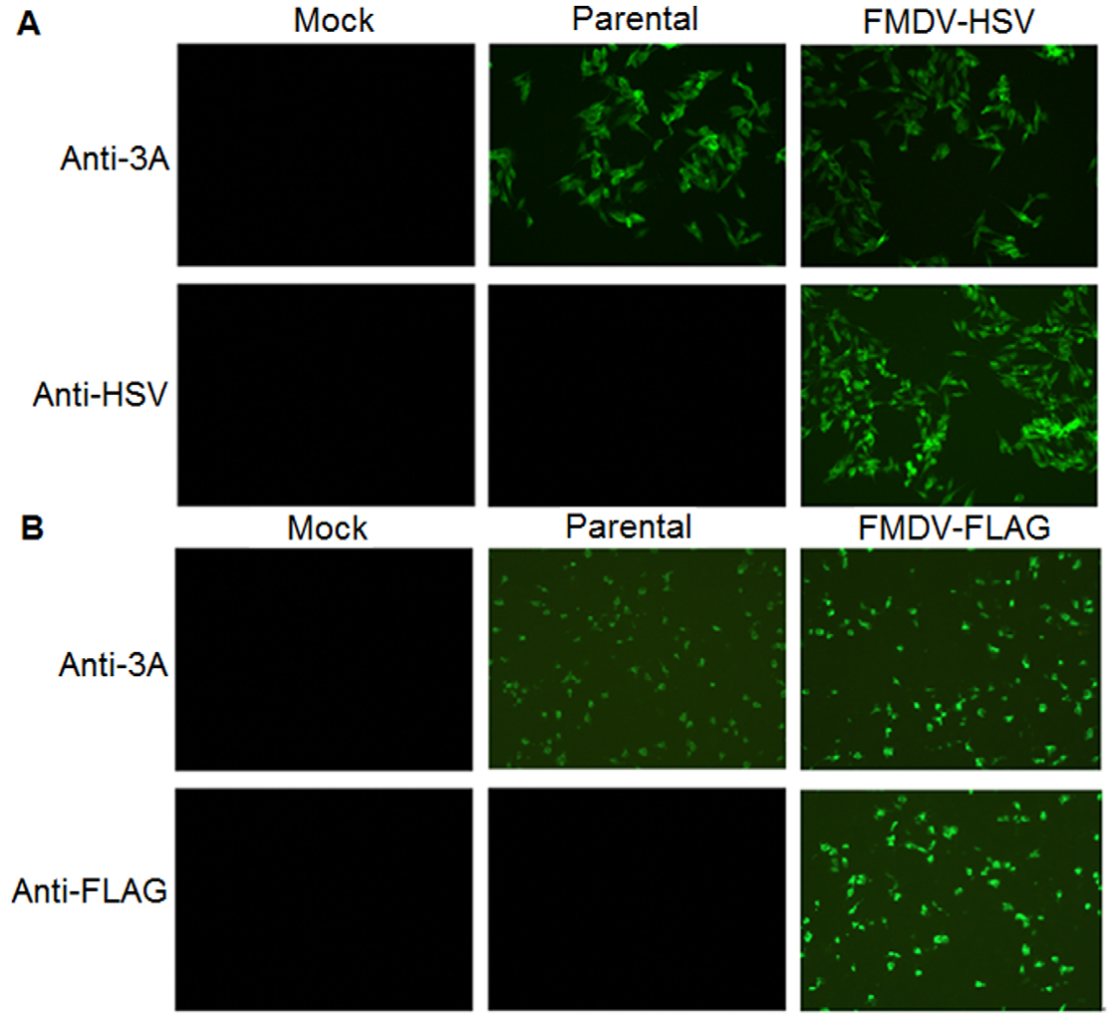
Immunofluorescence analysis of the expression of epitope tags in recombinant viruses. Confluent BHK-21 cells were mock-infected or infected with r-HN or the transfected supernatants at a MOI of 1, incubated for 6 h, fixed and probed with anti-3A and anti-HSV (A) or anti-FLAG mAb (B), followed by incubation with FITC-conjugated secondary antibody. The cells were visualized under an Olympus BX40 fluorescence microscope. Magnification, ×10.

**Figure 3:**
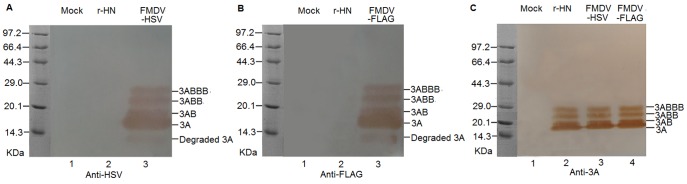
Western blot analysis of the expression of epitope tags in recombinant viruses. BHK-21 cells were mock-infected or infected with r-HN or the transfected supernatants at a MOI of 1 and incubated at 37°C. Infected cell extracts were prepared at 12 h post-infection. Proteins were separated on a 12% SDS-PAGE, blotted, and probed with anti-HSV(A), anti-FLAG (B) or anti-3A mAb (C). The numbers on the left represent molecular mass standards in kilodaltons (kDa).

To determine whether the recombinant viruses express the foreign tag proteins, BHK-21 cells were mock-infected or infected with r-HN or the transfected supernatants at a MOI of 1. At 6 hpi, the cells were fixed and incubated with mouse anti-3A and anti-HSV or anti-FLAG mAbs, followed by immunostaining with FITC-conjugated goat anti-mouse IgG. The results showed that the anti-HSV mAb readily detected viral antigens in FMDV-HSV-infected cells but not in mock-infected or rHN-infected controls (Fig. 2A). Similarly, the FLAG tag protein of FMDV-FLAG was detected by anti-FLAG mAb, whereas no signal was seen in mock-infected or rHN-infected controls (Fig. 2B). In contrast, the anti-3A mAb readily detected viral antigens in r-HN, FMDV-FLAG, or FMDV-HSV-infected cells but not in mock-infected cells (Fig. 2A and Fig. 2B). To further confirm the expression of tag proteins by the recombinant viruses, mock, r-HN or 3A-tagged virus-infected BHK-21 cell lysates were harvested at 12 hpi, and cytoplasmic proteins were resolved by SDS-PAGE and analyzed by Western blotting. As expected, all 3A-containing bands were detected in recombinant virus-infected cell lysates with anti-tag antibodies, but not in mock-infected cells. As shown in Figure 3A and Figure 3B, the fastest migrating band represents degraded-3A [12] and the remaining four bands correspond to 3A, 3AB, 3ABB and 3ABBB. Furthermore, 3A-containing protein bands were also clearly detected in FMDV-HSV, FMDV-FLAG or r-HN-infected cell lysates using anti-3A mAb, but not in mock-infected cells (Fig. 3C). The degraded-3A was not visible in this assay. Taken together, these results suggested that the tag genes were stably maintained and expressed during a large number of virus generations in cell culture.

### RT-PCR Analysis of Recombinant Viruses

**Figure 4:**
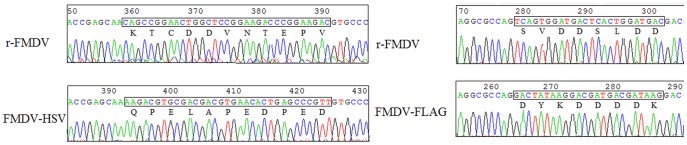
Electrophoregram of the partial 3A gene of the parental virus in comparison with the modified 3A gene of the recombinant viruses. The modified 3A genes derived from cell-culture passage 11 (P11) of FMDV-HSV (A) and FMDV-FLAG (B) were sequenced. Amino acids are shown as the single-letter code and the nucleotide sequences of tags are boxed.

To confirm the rescued viruses were the correct recombinants, viral RNAs were extracted from the transfected supernatants and analyzed by RT-PCR using primers HN-1F and HN-2R. As expected, the fragments of 1050 bp were obtained from the transfected supernatants. In contrast, PCR of the same viral RNA without RT failed to produce any product (data not shown), indicating that the PCR products originated from viral RNA but not from the transfected plasmids. The amplicons were sequenced to confirm the rescued viruses contained expected modification. To determine the genetic stability of the recombinant viruses *in* *vitro*, the transfected supernatants were serially passaged in BHK-21 cells 11 times, and the presence of the tag genes in the recombinant viruses from 4th, 8th and 11th passage was analyzed by RT-PCR and sequencing. The results showed that the sequence identities of the tag genes were preserved (Fig. 4A and Fig. 4B). At the same time, IFA and Western blot analysis of the recombinant viruses (p11) also provided positive identification of the HSV and FLAG tags. Take together, these results suggested that FMDV can stably sustain the insertion of small foreign tags in 3A protein even after 11 serial passages in BHK-21 cells.

### Growth Characteristics of the Recombinant Viruses

**Figure 5:**
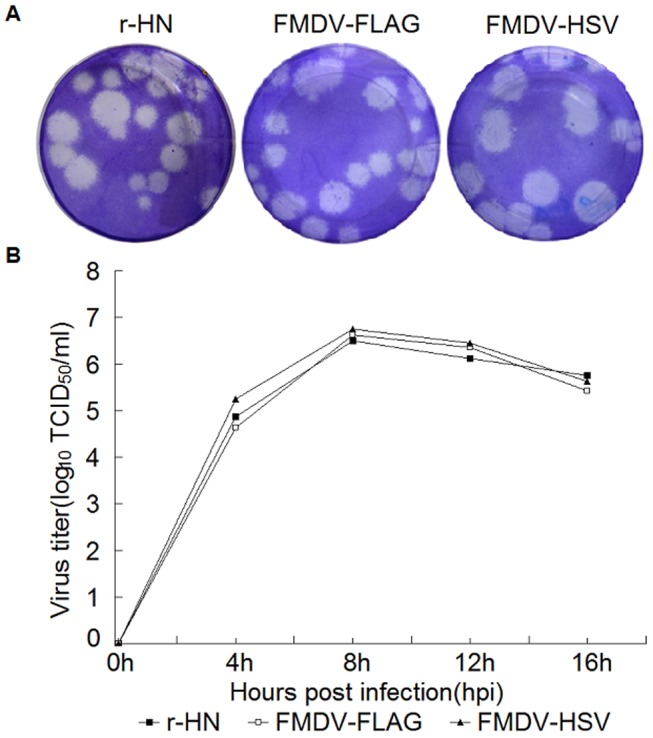
Plaque morphology and growth curves of the parental virus and the 3A-tagged viruses in BHK-21 cells. (A) Plaque morphology of the parental virus and the 3A-tagged viruses in BHK-21 cells. Viruses were plaque-assayed under tragacanth gum on BHK-21 cells and cells were stained at 48 h hpi. Plaques from appropriate dilutions are shown. (B) Growth curves of the parental virus and the 3A tagged viruses in BHK-21 cells. BHK-21 cells were infected with the parental virus or the 3A-tagged viruses at a MOI of 1. At 0, 4, 8, 12 and 16 hpi, cells and supernatants were harvested and virus titers were determined by TCID_50_/ml on BHK-21 cells. The values of the viral titer represent the average obtained from triplicate experiments.

The effects of the tag introductions into 3A protein of FMDV on the growth characteristics were compared with those of the parental virus. As shown in Figure 5A, three viruses produced mixed plaques (containing large, medium and small plaques) and there were no significant differences in the plaque size and morphology between the 3A tagged viruses and the parental virus. The single-step growth kinetics of the mutant viruses and the parental virus in BHK-21 cells were also very similar (Fig.5B), though the titers of FMDV-HSV were slightly higher than parental virus and FMDV-FLAG at 4 h, 8 h and 12 h. These results indicated that introduction of the foreign epitopes into the 3A genome of FMDV did not significantly influence the virus growth characteristics in cell culture.

### Detection of Anti-tag Antibodies in Mice

To determine the presence of antibodies specific for the introduced HSV or FLAG epitopes, groups of six-week-old Kunming mice were intraperitoneally inoculated with r-HN, FMDV-HSV, or FMDV-FLAG. Four weeks after inoculation, blood samples were analyzed with ELISA. The results showed that two mice in each group infected with FMDV-HSV or FMDV-FLAG viruses developed anti-tag antibodies and anti-3ABC antibodies (Table 3), and the antibody titers of anti-tag and anti-3ABC were highly correlated. Two mice infected with parental virus only produced anti-3ABC antibodies (Table 3). These results indicated that the 3A-tagged recombinant viruses or the parental virus could only replicate in some mice after inoculation. The reason for this phenomenon is unknown. The serological results showed that 3A-tagged viruses were able to induce tag specific antibody responses by 28 dpi that was distinguishable from the antibody response induced by the parental virus.

**Table 3:** The OD_450_ value of anti-tag and anti-3ABC antibodies in mice infected with the 3A-tagged viruses and the parental virus.

Virus	Animal No.	3ABC	HSV	FLAG
FMDV-HSV	M1	1.495(+)	1.665(+)	/
	M2	0.267(−)	0.282(−)	/
	M3	1.565(+)	1.623(+)	/
	M4	0.288(−)	0.308(+)	/
	M5	0.271(−)	0.295(−)	/
	M6	0.316(−)	0.365(+)	/
FMDV-FLAG	M7	1.622(+)	/	1.517(+)
	M8	0.248(+)	/	0.272(−)
	M9	0.127(−)	/	0.134(−)
	M10	0.309(−)	/	0.320(−)
	M11	1.679(+)	/	1.868(+)
	M12	0.255(−)	/	0.295(−)
r-HN	M13	1.695(+)	0.265(−)	0.237(−)
	M14	0.267(−)	0.282(−)	0.203(−)
	M15	1.565(+)	0.223(−)	0.166(−)
	M16	0.288(−)	0.308(−)	0.240(−)
	M17	0.271(−)	0.295(−)	0.272(−)
	M18	0.316(−)	0.365(−)	0.248(−)
Mean Neg.Control(n = 4)		0.308	0.272	0.255
Mean Pos. Control		1.445	/	/
Cutoff value		0.525	0.571	0.536

The cutoff value of tag antibodies = 2.1×mean negative control. The cutoff value of 3ABC antibodies was calculated as previously described [37].

+, represents antibody positive; −, represent antibody negative.

## Discussion

The development of viral vectors is a relatively novel and promising approach to study virus replication, gene function, virus pathogenesis and to develop marker vaccines as well as gene therapy. However, identifying acceptable sites in the viral genome for insertion of foreign sequences to generate viable viruses can be technically very challenging. Genetic comparisons of the 3A proteins of different FMDV isolates revealed that the first half of the 3A coding region, which encodes an N-terminal hydrophilic domain, is highly conserved, however, the latter half of the 3A coding region (the C-terminal domain) is substantially variable in sequence [40], [41] and can naturally incorporate different deletions (including residues 85–102, 93–102 and 133–143 deletions) [8], [9], [12], [41]. The 93–102 deletion in 3A was responsible for its attenuation in bovines but remain highly pathogenic in pigs [8], [42]. Furthermore, previous study showed that FMDV expressing a full-length 3A can tolerate artificial deletion (93–102 aa) and did not affect the ability of virus replication *in vitro* [43]. Based on these properties, we reasoned that the regions of 85–102 and 133–143 of 3A are non-essential regions for FMDV replication and are suitable targets for manipulation to insert foreign epitopes. In the case of O/HN/CHA/93, the deletion occurs at positions 93 to 102 aa of the 3A protein [44]. Therefore, in this study, two regions (85–92 and 133–143) of 3A were chosen for individual epitope tag insertions to study the ability of FMDV expressing foreign epitope tags.

A major obstacle common to the proposed RNA virus expression vector is their inherent genetic instability. Especially, the viruses expressing some large foreign proteins were readily lost parts or the entirety during recombinant virus replication in cell culture [26], [45], [46]. This tendency may be simply explained by the deleterious effect of the insertion of foreign sequences on virus replication efficiency, triggering adaptation to a faster growing phenotype. In view of these complications, in this study, we used an 8 aa FLAG epitope and an 11 aa HSV epitope to replace the native amino acid sequences of 85–92 and 133–143 regions of the 3A protein respectively, which did not increase the genome length. In addition, HSV and FLAG epitope tags were selected because they are well characterized and are recognized by commercial monoclonal antibodies.

In the present study, we explored the potential utility of FMDV to carry foreign epitopes with the aim of developing a safe and efficient viral vector platform. Using previously established FMDV reverse genetics system [33], FLAG and HSV tags were introduced into the 85–92 and 133–143 regions of the 3A protein respectively. As expected, two viable viruses were recovered after transfection of two modified constructs into BSR/T7 cells, suggesting that FMDV 3A could tolerate introduction of the foreign epitopes into its C terminus and retain functionality in FMDV replication. IFA, Western blot and sequence analysis showed that the 3A-tagged viruses stably maintained the foreign epitopes even after 11 serial passages in BHK-21. Comparison of the recombinant viruses expressing foreign epitopes and the parental virus revealed that the viruses behaved similarly in cell culture with regards to plaque morphology and growth kinetics. This result is consistent with previous report that the insertion of foreign epitopes in 3A protein of PV, did not affect recombinant PVs growth properties and characteristics [39].

The 3A-tagged viruses experimentally infected Kunming mice, no clinical signs were observed in the virus-infected mice and control mice during the entire experimental process. After four weeks post-infection, some mice inoculated with 3A-tagged viruses could induce both anti-tag and anti-3ABC antibodies, however, some mice infected with the parental virus only produced anti-3ABC antibody. The serological results showed that 3A-tagged viruses were able to induce specific antibody response that was distinguishable from the antibody response induced by the parental virus. Therefore, the 3A-tagged viruses could potentially serve as marker vaccine to allow the serologic differentiation of vaccinated animals from naturally infected ones.

Recently, the other advances in protein purification [47], intracellular location [48], examination of the role of the viral protein in the virus life cycle [48], study of interactions of virus protein with viral and cellular protein in infected cells [39] as well as investigation of virus structure and morphogenesis [49] often rely on the small tag-expressing viruses. To date, the functions of nonstructural protein 3A in the life cycle of FMDV, subcellular distribution of FMDV 3A protein containing 93–102 aa deletion in pig and bovine as well as the interactions of FMDV protein 3A with other viral and cellular factors in infected cells are poorly understood. Therefore, this work would represent useful tools to monitor subcellular localization and dynamics during FMDV infection and to determine the interactions of 3A protein with other viral and cellular factors during virus replication in infected cells.

In conclusion, this is the first report describing introduction of foreign epitope tags into FMDV protein 3A and generation of recombinant viruses that stably express small epitope tags. Further study is in progress to investigate the maximum size of foreign sequences that the 3A genome of FMDV can tolerate without loosing infectivity.

## References

[1] Porter AG (1993) Picornavirus nonstructural proteins: emerging roles in virus replication and inhibition of host cell functions. J Virol 67: 6917–6921. 8230412 10.1128/jvi.67.12.6917-6921.1993 PMC238148

[2] Belsham GJ (1993) Distinctive features of foot-and-mouth disease virus, a member of the picornavirus family: aspects of virus protein synthesis, protein processing and structure. Prog Biophys Mol Biol 60: 241–260. 8396787 10.1016/0079-6107(93)90016-D PMC7173301

[3] Bienz K, Egger D, Pasamontes L (1987) Association of polioviral proteins of the P2 genomic region with the viral replication complex and virus induced membrane synthesis as visualized by electron microscopic immunocytochemistry and autoradiography. Virology 160: 220–226. 2820130 10.1016/0042-6822(87)90063-8

[4] Bienz K, Egger D, Rasser Y, Bossart W (1983) Intracellular distribution of poliovirus proteins and the induction of virus-specific cytoplasmic structures. Virology 131: 39–48. 6316654 10.1016/0042-6822(83)90531-7

[5] Schlegel A, Giddings TH, Ladinsky JMS, Kirkegaard K (1996) Cellular origin and ultrastructure of membranes induced during poliovirus infection. J Virol 70: 6576–6588. 8794292 10.1128/jvi.70.10.6576-6588.1996 PMC190698

[6] Towner JS, Ho TV, Semler BL (1996) Determinants of membrane association for poliovirus protein 3AB. J Biol Chem 271: 26810–26818. 8900162 10.1074/jbc.271.43.26810

[7] Giraudo AT, Beck E, Strebel K, Auge de Mello P, Torre JL, et al (1990) Identification of a nucleotide deletion in parts of polypeptide 3A in two independent attenuated apthovirus strains. Virology 177: 780–788. 2164734 10.1016/0042-6822(90)90549-7

[8] Beard CW, Mason PW (2000) Genetic determinants of altered virulence of Taiwanese foot-and-mouth disease virus. J Virol 74: 987–991. 10623761 10.1128/jvi.74.2.987-991.2000 PMC111619

[9] Knowles NJ, Davies PR, Henry T, O’Donnell V, Pacheco JM, et al (2001) Emergence in Asia of foot-and-mouth disease viruses with altered host range: characterization of alteration s in the 3A protein. J Virol 75: 1551–1556. 11152528 10.1128/JVI.75.3.1551-1556.2001 PMC114061

[10] Nunez JI, Baranowski E, Molina N, Jarabo RCM, Sanchez C, et al (2001) A single amino acid substitution in nonstructural protein 3A can mediate adaptation of foot-and-mouth disease virus to the guinea pig. J Virol 75: 3977–3983. 11264387 10.1128/JVI.75.8.3977-3983.2001 PMC114889

[11] Nunez JI, Molina N, Baranowski E, Domingo E, Clark S, et al (2007) Guinea pig-adapted foot-and-mouth disease virus with altered receptor recognition can productively infect a natural host. J Virol 81: 8497–8506. 17522230 10.1128/JVI.00340-07 PMC1951369

[12] Pacheco JM, Henry TM, O’Donnel VK, Gregory JB, Mason PW (2003) Role of nonstructural proteins 3A and 3B in host range and pathogenicity of foot-and-mouth disease virus. J Virol 77: 13017–13027. 14645558 10.1128/JVI.77.24.13017-13027.2003 PMC296074

[13] Bukreyev A, Camargo E, Collins PL (1996) Recovery of infectious respiratory syncytial virus expressing an additional, foreign gene. J Virol 70: 6634–6641. 8794298 10.1128/jvi.70.10.6634-6641.1996 PMC190704

[14] Mebatsion T, Schnell MJ, Cox JH, Finke S, Conzelmann KK (1996) Highly stable expression of a foreign gene from rabies virus vectors. Proc Natl Acad Sci USA 93: 7310–7314. 8692989 10.1073/pnas.93.14.7310 PMC38980

[15] Schnell MJ, Buonocore L, Whitt MA, Rose JK (1996) Theminimal conserved transcription stop-start signal promotes stable expression of a foreign gene in vesicular stomatitis virus. J Virol 70: 2318–2323. 8642658 10.1128/jvi.70.4.2318-2323.1996 PMC190073

[16] Engel-Herbert I, Werner O, Teifke JP, Mebatsion T, Mettenleiter TC, et al (2003) Characterization of a recombinant Newcastle disease virus expressing the green fluorescent protein. J Virol Methods 108: 19–28. 12565150 10.1016/s0166-0934(02)00247-1

[17] Singh M, Cattaneo R, Billeter MA (1999) A recombinant measles virus expressing hepatitis B virus surface antigen induces humoral immune responses in genetically modified mice. J Virol 73: 4823–4828. 10233943 10.1128/jvi.73.6.4823-4828.1999 PMC112525

[18] Groot Bramel-Verheije MH, Rottier PJ, Meulenberg JJ (2000) Expression of a foreign epitope by porcine reproductive and respiratory syndrome virus. Virology 278: 380–389. 11118361 10.1006/viro.2000.0525

[19] Andino R, Silvera D, Suggett SD, Achacoso PL, Miller CJ, et al (1994) Engineering poliovirus as a vaccine vector for the expression of diverse antigens. Science 265: 1448–1451. 8073288 10.1126/science.8073288

[20] Kim DS, Cho YJ, Kim BG, Lee SH, Nam JH (2010) Systematic analysis of attenuated Coxsackievirus expressing a foreign gene as a viral vaccine vector. Vaccine 28: 1234–1240. 19941986 10.1016/j.vaccine.2009.11.017

[21] Hammoumi S, Cruciere C, Guy M, Boutrouille A, Messiaen S, et al (2006) Characterization of a recombinant encephalomyocarditis virus expressing the enhanced green fluorescent protein. Arch Virol 151: 1783–1796. 16575480 10.1007/s00705-006-0746-7

[22] Zhang L, Sato S, Kim JI, Roos RP (1995) Theiler’s Virus as a Vector for Foreign Gene Delivery. J Virol 69: 3171–3175. 7707546 10.1128/jvi.69.5.3171-3175.1995 PMC189020

[23] Arnold GF, Resnick DA, Li Y, Zhang A, Smith AD, et al (1994) Design and construction of rhinovirus chimeras incorporating immunogens from polio, influenza, and human immunodeficiency viruses. Virology 198: 703–708. 7507283 10.1006/viro.1994.1082

[24] Beard MR, Cohen L, Lemon SM, Martin A (2001) Characterization of Recombinant Hepatitis A Virus Genomes Containing Exogenous Sequences at the 2A/2B Junction. J Virol 75: 1414–1426. 11152515 10.1128/JVI.75.3.1414-1426.2001 PMC114048

[25] Natalya LT, Levenson EA, Ehrenfeld E (2009) Viable Polioviruses That Encode 2A Proteins with Fluorescent Protein Tags. J Virol 84: 1477–1488. 19939919 10.1128/JVI.01578-09 PMC2812313

[26] Chapman NM, Kim KS, Tracy S, Jackson J, Hofling K, et al (2000) Coxsackievirus expression of the murine secretory protein interleukin-4 induces increased synthesis of immunoglobulin G1 in mice. J Virol 74: 7952–7962. 10933703 10.1128/jvi.74.17.7952-7962.2000 PMC112326

[27] Mattion NM, Reilly PA, DiMichele SJ, Crowley JC, Weeks-Levy C (1994) Attenuated poliovirus strain as a live vector: expression of regions of rotavirus outer capsid protein VP7 by using recombinant Sabin 3 viruses. J Virol 68: 3925–3933. 8189529 10.1128/jvi.68.6.3925-3933.1994 PMC236898

[28] Mattion NM, Reilly PA, Camposano E, Wu SL, DiMichele SJ, et al (1995) Characterization of recombinant polioviruses expressing regions of rotavirus VP4, hepatitis B surface antigen, and herpes simplex virus type 2 glycoprotein D. J Virol. 69: 513–527. 10.1128/jvi.69.8.5132-5137.1995 PMC189333 7609083

[29] Mueller S, Wimmer E (1998) Expression of foreign proteins by poliovirus polyprotein fusion: analysis of genetic stability reveals rapid deletions and formation of cardioviruslike open reading frames. J Virol 72: 20–31. 9420196 10.1128/jvi.72.1.20-31.1998 PMC109345

[30] Piccone ME, Diaz-San Segundo F, Kramer E, Rodriguez LL, de los Santos T (2011) Introduction of tag epitopes in the inter-AUG region of foot and mouth disease virus: Effect on the L protein. Virus Research 155: 91–97. 20849893 10.1016/j.virusres.2010.09.004

[31] Rieder E, Bunch T, Brown F, Mason PW (1993) Genetically engineered foot-and-mouth disease viruses with poly(C) tracts of two nucleotides are virulent in mice. J Virol 67: 5139–5145. 8394441 10.1128/jvi.67.9.5139-5145.1993 PMC237911

[32] Buchholz UJ, Finke S, Conzelmann KK (1999) Generation of bovine respiratory syncytial virus (BRSV) from cDNA: BRSV NS2 is not essential for virus replication in tissue culture, and the human RSV leader region acts as a functional BRSV genome promoter. J Virol 73: 251–259. 9847328 10.1128/jvi.73.1.251-259.1999 PMC103829

[33] Li PH, Bai XW, Sun P, Li D, Lu ZJ, et al. (2012) Evaluation of a genetically modified foot-and-mouth disease virus vaccine candidate generated by reverse genetics. BMC Veterinary Research. doi:10.1186/1746-6148-8-57. 10.1186/1746-6148-8-57 PMC3488552 22591597

[34] Wu L, Tian MN, Lu ZJ, Fu YF, Sun P, et al (2010) Preparation and identification of monoclonal antibodies against nonstructural protein 3A of foot-and-mouth disease virus. Chinese Veterinary Science 40: 331–336.

[35] Sambrook J, Fitsch EF, Maniatis T (1989) Molecular Cloning: A Laboratory Manual. Cold Spring Harbor: Cold Spring Harbor Press.

[36] Rieder E, Bunch T, Brown F, Mason PW (1993) Genetically engineered foot-and-mouth disease viruses with poly(C) tracts of two nucleotides are virulent in mice. J Virol 67: 5139–5145. 8394441 10.1128/jvi.67.9.5139-5145.1993 PMC237911

[37] Lu Z, Cao Y, Guo J, Qi S, Li D, et al (2007) Development and validation of a 3ABC indirect ELISA for differentiation of foot-and-mouth disease virus infected from vaccinated animals. Vet Microbiol 125(1–2): 157–169. 17601688 10.1016/j.vetmic.2007.05.017

[38] Natalya LT, Chris L, Kenneth SJ, Eric AL, Alexander EG, et al (2011) Identification of tolerated insertion sites in poliovirus non-structural proteins. Virology 409: 1–11. 20971490 10.1016/j.virol.2010.09.028 PMC2993843

[39] Teterina NL, Pinto Y, Weaver JD, Jensen KS, Ehrenfeld E (2011) Analysis of Poliovirus Protein 3A Interactions with Viral and Cellular Proteins in Infected Cells. J Virol 85: 4284–4296. 21345960 10.1128/JVI.02398-10 PMC3126254

[40] Maroudam V, Nagendrakumar SB, Rangarajan PN, Thiagarajan D, Srinivasan VA (2010) Genetic characterization of Indian type O FMD virus 3A region in context with host cell preference. Infection, Genetics and Evolution 10: 703–709. 10.1016/j.meegid.2010.03.004 20302973

[41] Jajati KM, Aniket S, Divakar H, Chakradhar T, Subhajit B, et al (2008) Comparative genomics of serotype Asia 1 foot-and-mouth disease virus isolates from India sampled over the last two decades. Virus Research 136: 16–29. 18511143 10.1016/j.virusres.2008.04.010

[42] O’Donnell VK, Pacheco JM, Henry TM, Mason PW (2001) Subcellular distribution of the foot-and-mouth disease virus 3A protein in cells infected with viruses encoding wild-type and bovine-attenuated forms of 3A. Virology 287: 151–162. 11504550 10.1006/viro.2001.1035

[43] Li S, Gao M, Zhang R, Song G, Song J, et al (2010) A mutant of infectious Asia 1 serotype foot-and-mouth disease virus with the deletion of 10-amino-acid in the 3A protein. Virus genes 41: 406–413. 20844943 10.1007/s11262-010-0529-9

[44] Samuel AR, Knowles NJ (2001) Foot-and-mouth disease type O viruses exhibit genetically and geographically distinct evolutionary lineages (topotypes). J Gen Virol 82: 609–621. 11172103 10.1099/0022-1317-82-3-609

[45] Mueller S, Wimmer E (1998) Expression of foreign proteins by poliovirus polyprotein fusion: analysis of genetic stability reveals rapid deletions and formation of cardioviruslike open reading frames. J Virol 72: 20–31. 9420196 10.1128/jvi.72.1.20-31.1998 PMC109345

[46] Tang S, van Rij R, Silvera D, Andino R (1997) Toward a poliovirus-based simian immunodeficiency virus vaccine: correlation between genetic stability and immunogenicity. J Virol 71: 7841–7850. 9311872 10.1128/jvi.71.10.7841-7850.1997 PMC192139

[47] Wegelt A, Reimann I, Granzow H, Beer M (2011) Characterization and purification of recombinant bovine viral diarrhea virus particles with epitope-tagged envelope proteins. J Gen Virol 92 (Pt 6): 1352–7. 10.1099/vir.0.029330-0 21346033

[48] Benjamin B, Ping L, Richard E (2011) Generation and characterisation of a recombinant Rift Valley fever virus expressing a V5 epitope-tagged RNA-dependent RNA polymerase. J Gen Virol 92: 2906–2913. 21900422 10.1099/vir.0.036749-0

[49] Joanne LT, Norihito U, Andrew AM, Stephen BF (2009) Investigation of orf virus structure and morphogenesis using recombinants expressing FLAG-tagged envelope structural proteins: evidence for wrapped virus particles and egress from infected cells. J Gen Virol 90: 614–625. 19218206 10.1099/vir.0.005488-0

